# Multiple R2R3-MYB Transcription Factors Involved in the Regulation of Anthocyanin Accumulation in Peach Flower

**DOI:** 10.3389/fpls.2016.01557

**Published:** 2016-10-21

**Authors:** Hui Zhou, Qian Peng, Jianbo Zhao, Albert Owiti, Fei Ren, Liao Liao, Lu Wang, Xianbao Deng, Quan Jiang, Yuepeng Han

**Affiliations:** ^1^Key Laboratory of Plant Germplasm Enhancement and Specialty Agriculture, Wuhan Botanical Garden of the Chinese Academy of SciencesWuhan, China; ^2^University of Chinese Academy of SciencesBeijing, China; ^3^Institute of Forestry and Pomology, Beijing Academy of Agriculture and Forestry SciencesBeijing, China; ^4^Sino-African Joint Research Center, Chinese Academy of SciencesWuhan, China

**Keywords:** peach, anthocyanin, proanthocyanin, flower coloration, R2R3-MYB

## Abstract

Anthocyanin accumulation is responsible for flower coloration in peach. Here, we report the identification and functional characterization of eight flavonoid-related R2R3-MYB transcription factors, designated *PpMYB10.2, PpMYB9, PpMYBPA1, Peace, PpMYB17, PpMYB18, PpMYB19*, and *PpMYB20*, respectively, in peach flower transcriptome. *PpMYB10.2* and *PpMYB9* are able to activate transcription of anthocyanin biosynthetic genes, whilst *PpMYBPA1* and *Peace* have a strong activation on the promoters of proanthocyanin (PA) biosynthetic genes. *PpMYB17-20* show a strong repressive effect on transcription of flavonoid pathway genes such as *dihydroflavonol 4-reductase*. These results indicate that anthocyanin accumulation in peach flower is coordinately regulated by a set of R2R3-MYB genes. In addition, *PpMYB9* and *PpMYB10.2* are closely related but separated into two groups, designated MYB9 and MYB10, respectively. *PpMYB9* shows a strong activation on the *PpUGT78A2* promoter, but with no effect on the promoter of *PpUGT78B* (commonly called *PpUFGT* in previous studies). In contrast, *PpMYB10.2* is able to activate the *PpUFGT* promoter, but not for the *PpUGT78A2* promoter. Unlike the *MYB10* gene that is universally present in plants, the *MYB9* gene is lost in most dicot species. Therefore, the *PpMYB9* gene represents a novel group of anthocyanin-related MYB activators, which may have diverged in function from the *MYB10* genes. Our study will aid in understanding the complex mechanism regulating floral pigmentation in peach and functional divergence of the R2R3-MYB gene family in plants.

## Introduction

Flower coloration is one of the most important traits in ornamental plants. In general, pigmentation in flowers is due to accumulation of various classes of pigments, such as anthocyanins, chlorophylls, carotenoids, and betalains (Tanaka et al., 2008). Anthocyanins are a kind of widely distributed water-soluble pigments, which are ultimately synthesized from phenylalanine in the cytosolic surface of the endoplasmic reticulum and stored in the vacuole. Anthocyanins are glycosylated forms of anthocyanidins catalyzed by glycosyltransferase in species-specific manner, with the sugar moiety predominantly attached at the 3-position on the C-ring or the 5-position on the A-ring (Caputi et al., 2012). Although more than 550 anthocyanins have been identified in nature, there are only six major types of anthocyanidins: pelargonidin, cyanidin, peonidin, delphinidin, petunidin, and malvidin (Kong et al., 2003). The color change of anthocyanidins is mainly attributed to the number of hydroxyl groups on the B-ring, with more hydroxyl groups shifting the color to blue. Glycosylation of anthocyanidins has a slight reddening effect (Tanaka et al., 2008). Thus, understanding mechanism regulating anthocyanin biosynthesis is crucial for genetic manipulation of flower coloration in ornamental plants.

The anthocyanin biosynthetic pathway has been elucidated in a variety of plant species (Grotewold, 2006). This pathway involves a number of reactions, which are catalyzed by various enzymes such as chalcone synthase (CHS), chalcone isomerase (CHI), flavanone 3-hydroxylase (F3H), flavonoid 3′ hydroxylase (F3′H), flavonoid 3′,5′-hydroxylase (F3′5′H), dihydroflavonol 4-reductase (DFR), leucoanthocyanidin dioxygenase (LDOX), and UDP-glucose flavonoid 3-*O*-glucosyltransferase (UFGT). Of these enzymes, F3′5′H is absent in some plants such as *Arabidopsis* (Forkmann, 1991), apple (Han et al., 2010), and peach (Cheng et al., 2014), and each is encoded by gene families. For example, two *UFGT78* genes, *PpUGT78A2* and *PpUGT78B*, are identified in the peach genome, and both of them can catalyze the glycosyl transfer on the C3 position of the anthocyanidin aglycone in flower (Cheng et al., 2014).

Besides anthocyanin pathway structural genes, various regulatory genes, such as MYB, basic helix-loop-helix (bHLH), and WD40 transcription factors (TFs), are also involved in anthocyanin biosynthesis. Although these three types of TFs form a complex to coordinately regulate the transcription of anthocyanin pathway structural genes, MYB TFs such as *Arabidopsis* AtPAP1/AtMYB75 (Borevitz et al., 2000) or its homologs are widely reported to play a determinant role in anthocyanin coloration in various dicotyledonous plants, such as *Antirrhinum majus* (Schwinn et al., 2006), cauliflower (Chiu et al., 2010), Petunia (Albert et al., 2011), kiwifruit (Fraser et al., 2013), Asiatic hybrid lily (Yamagishi et al., 2014), monkeyflowers (Yuan et al., 2014), and *Phalaenopsis* spp. (Hsu et al., 2015).

Peach belongs to the genus *Pruns* in the Rosaceae family. It is a diploid, with a basic chromosome number of *x* = 8. Peach has a small genome of approximately 230 Mb per haploid and its draft genome sequence was released in 2010 (Verde et al., 2013). More recently, several anthocyanin-related MYB genes have been identified to control anthocyanin accumulation in peach. For example, three R2R3-MYB genes, *PpMYB10.1, PpMYB10.*2, and *PpMYB10.*3, are involved in anthocyanin accumulation in fruit (Ravaglia et al., 2013; Rahim et al., 2014; Zhou et al., 2015b), while the *PpMYB10.4* gene is responsible for red coloration of leaf (Zhou et al., 2014). A TT2-like R2R3-MYB gene, designated *Peace*, regulates petal pigmentation in flowering peach ‘Genpei’ bearing variegated and fully pigmented flowers although it is unclear whether the *Peace* gene is able to activate anthocyanin and/or proanthocyanin pathway structural genes (Uematsu et al., 2014). However, our study shows that a small indel mutation in an anthocyanin transporter, which is not the result of a transposon insertion or excision, causes variegated colouration of flowers of ornamental peach, ‘Hongbaihuatao’ (Cheng et al., 2015). These results suggest a complex mechanism regulating anthocyanin accumulation in peach flower.

We previously conducted RNA-Seq for different tissues of peach, including leaf, flower, and fruit (Wang et al., 2013). Here, we analyzed the RNA-Seq transcriptome of peach flower, and a total of eight flavonoid-related R2R3-MYB TFs, designated *PpMYB10.2, PpMYB9, PpMYBPA1, Peace, PpMYB17, PpMYB18, PpMYB19*, and *PpMYB20*, were identified. Among these MYB TFs, *PpMYB10.2*, and *PpMYB9* are anthocyanin activators, while *PpMYBPA1* and *Peace* are involved in the regulation of proanthocyanin (PA) biosynthesis. The rest four MYBs are flavonoid repressors. Interestingly, the *PpMYB9* gene showed the highest level of expression in flower and it represents a novel R2R3-MYB family regulating specific *UFGT* gene in peach. Our study reveals a set of R2R3-MYBs involved in the regulation of anthocyanin accumulation in peach flower, which will be helpful for manipulating anthocyanin coloration in peach programs in the future.

## Materials and Methods

### Plant Materials

Peach accessions used for transcriptome analysis in this study contained one cultivar Dahongpao and two ornamental cultivars, Hongbaihua and Hongyetao. These three cultivars are maintained at Wuhan Botanical Garden of the Chinese Academy of Sciences (Beijing, China). Blood-fleshed fruits of ‘Dahongpao,’ red flowers of ‘Hongbaihua,’ and red leaves of ‘Hongyetao’ were collected at 85 days after full-bloom (DAFB), flower bud stage, and juvenile stage in spring season, respectively. For cv. Mantianhong, flower samples were collected at flower bud, balloon and full-balloon stages, respectively. All the samples were immediately frozen in liquid nitrogen and then stored at -80°C until use.

### Identification of R2R3-MYB Genes in Peach Transcriptome

Details about cDNA library preparation, Illumina sequencing, and the mapping, assembly and GO (Gene Ontology) annotation of RNA-Seq data were present in our previous study (Wang et al., 2013). To validate the assembled unigenes, all unigenes were BLASTed against GenBank’s non-redundant protein (NR) and Swiss-Prot protein databases using BLASTx, and against GenBank’s non-redundant nucleotide (NT) database using BlastN, with an *E*-value cutoff of 1e-5. The FPKM values of R2R3-MYB genes showing high-level expression (FPKM ≥ 3) in one or more tissues tested were selected for further analysis. The FPKM value was used to create the heatmap using MeV (Multiple Experiment Viewer) 4.81 software (Howe et al., 2011).

### Construction of Expression Vectors

A pair of primers, MYB9F and MYB9R, was designed to amplify the full coding region of *PpMYB9* using KOD-Plus neo kit (Toyobo, Osaka, Japan). PCR products were purified, digested using restriction enzymes EcoRI and HindIII (New England Biolabs, Beverly, MA, USA), and inserted into the multiple cloning site (MCS) of pSAK277 binary vector. Similarly, the coding sequences of *PpMYB10.2, PpMYBPA1, Peace, PpMYB17, PpMYB18, PpMYB19*, and *PpMYB20* were cloned and inserted individually into the MCS of pSAK277, while the *PpbHLH3* gene was cloned and inserted into binary vector pHEX2 according to Zhou et al. (2015a). proUGT78A2::UGT78A2 was constructed in two steps: *UGT78A2* was inserted into the MCS of pSAK277 vector, followed with replacement of CaMV 35S promoter by the promoter of *PpUGT78A2* (1.8 Kb). The primers used for quantitative real-time PCR (qRT-PCR) are listed in Supplementary Table S1.

### Dual Luciferase Reporter Assay

A transient dual luciferase reporter assay was conducted in young leaf of tobacco (*Nicotiana benthamiana*) according to previously reported protocol (Hellens et al., 2005; Espley et al., 2007). Promoter regions upstream from the start codon of six peach genes, *DFR* (1.6 Kb), *LDOX* (1.9 Kb), *LAR1* (1.4 Kb), *ANR* (1.9 Kb), *UFGT* (2.5 Kb), and *UGT78A2* (1.8 Kb), were isolated and inserted individually into the MCS of vector pGreenII 0800-LUC. All the constructs were individually transformed into *A. tumefaciens* strain GV3101, and incubated at 28°C for 2 days. The confluent bacteria were resuspended in 10 ml infiltration buffer containing 10 mM MgCl_2_, 200 μM acetosyringone and 10 mM 2-(*N*-morpholine)-ethanesulfonic acid (pH = 5.7). Before infiltration, the bacteria were incubated at room temperature without shaking for 2 h. Transient transformation was conducted by mixing *Agrobacterium* GV3101 culture transformed with the reporter cassette and with the cassettes containing PpMYBs and PpbHLH3 driven by the CaMV 35S promoter with a ratio of 2:9:9. All tests were repeated at least four biological replicates. Firefly luciferase (Luc) and renilla luciferase (Ren) activity were measured 3 days after infiltration using Dual-Glo^®^ Luciferase Assay System (Promega, Madison, WI, USA) on an Infinite M200 luminometer (Tecan, Mannerdorf, Switzerland). Promoter activation activity was shown as the ratio of Luc activity to Renilla activity (Luc/Ren).

### Induction of Anthocyanins by Transient Infiltration in *Nicotiana tabacum* Leaf

Young tobacco seedlings (2–4 week old) were used for transient color assay. Agrobacteria cultivation and infiltration preparation were carried out following the same protocol as described above for transient dual luciferase assay. Equivalent doses of *PpMYB9, PpbHLH3*, and *proUGT78A2::UGT78A2* were mixed and infiltrated into the abaxial side of young leaves. The seedlings were placed in darkness overnight, and then moved to glasshouse under a long day photoperiod (16 h light/8 h dark). Photographs were taken at 2 weeks after infiltration.

### RNA Extraction and Quantitative Real-Time PCR (qRT-PCR)

Total RNA was extracted using Total RNA Rapid Extraction Kit (Zomanbio, Beijing, China). Removal of genomic DNA contamination and first strand cDNA synthesis were conducted using PrimeScript^TM^ RT reagent Kit with gDNA Eraser (Takara, Dalian, China). Quantitative RT-PCR was carried out in a total reaction volume of 20 μL containing 100 ng of template cDNA, 0.4 μM of each primer, 1 × ROX reference dye, and 10 μL of 2 × SYBR premix Ex Taq II (TaKaRa). The qRT-PCR program was as follows: one cycle of 30 s at 95°C, followed by 40 cycles of 5 s at 95°C and 34 s at 60°C. A translation elongation factor gene *PpTEF2* was selected as an internal control according to the previous report (Tong et al., 2009). All analyses were repeated three times using three biological replicates. The primers used for qRT-PCR are listed in Supplementary Table S2.

## Results

### Identification of Flavonoid-Related R2R3-MYB Transcription Factors in Peach Flower Transcriptome

**FIGURE 1:**
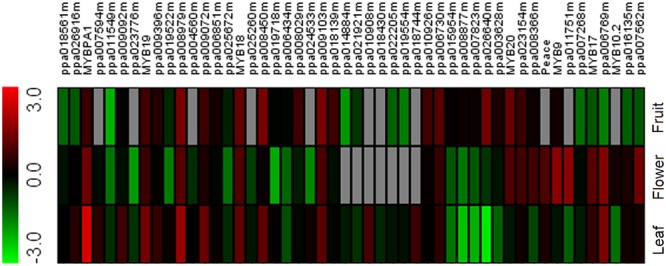
**Heatmap of R2R3-MYBs in peach transcriptomes of different red-colored tissues.** Green and red boxes indicate low- and high-expression levels, respectively. Red-colored leaf, flower, and fruit samples were collected from cv. Hongyetao, Hongbaihua, and Dahongpao, respectively.

In our previous study (Wang et al., 2013), we reported an RNA-Seq-based transcriptome database of peach. RNA-Seq data analysis revealed 148 transcripts encoding R2R3-MYB TFs. The expression level of these R2R3-MYB TFs was estimated in different tissues, including red flower, red leaf, and blood fruit. As a result, 49 R2R3-MYB TFs were identified to have relatively high expression levels (FPKM ≥ 3) in at least one of the three tissues tested. Subsequently, a heatmap representing their expression profiles was constructed based on the values of FPKM (fragments per kilobase of transcript per million mapped reads; **Figure 1**). Overall, the 49 R2R3-MYBs showed extensive variation in expression among the tested tissues. For example, *PpMYB10.1* (ppa026640m) was only expressed in blood mesocarp, while the transcript of *PpMYB10.4* (ppa018744m) was only observed in red leaf. Of the 49 R2R3-MYBs, eight, *PpMYB10.2* (ppa016711m), *PpMYB9* (ppa010069m), *PpMYBPA1* (ppa009439m), *Peace* (ppa023768m), *PpMYB17* (ppa010277m), *PpMYB18* (ppa010846m), *PpMYB19* (ppa010716m), and *PpMYB20* (ppa008906m), showed relatively high-level expression in flower. These eight R2R3-MYBs were selected and subjected to later analysis to elucidate mechanisms regulating anthocyanin biosynthesis in peach flower.

**FIGURE 2:**
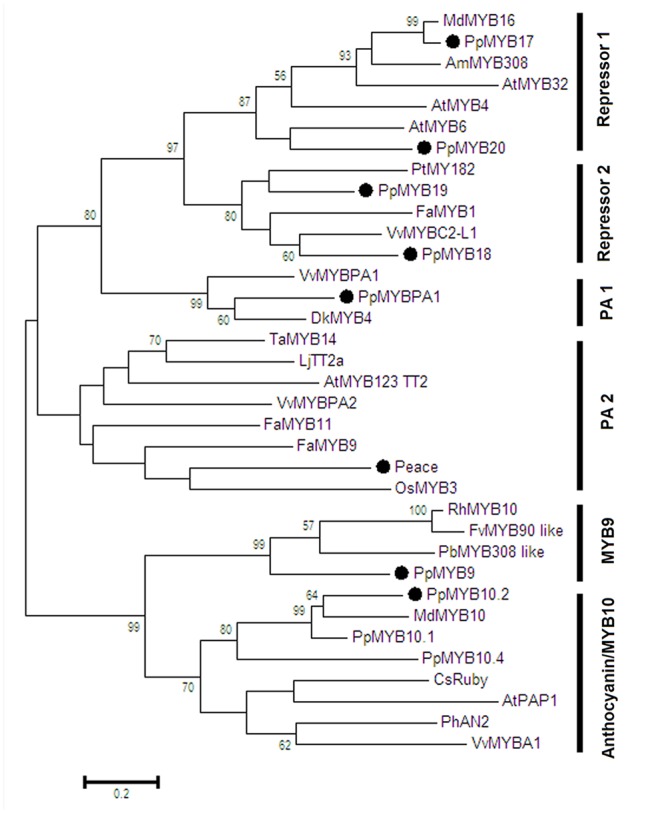
**Phylogenetic tree derived from amino acid sequences of R2R3-MYBs in peach and other dicot species.** The peach R2R3-MYBs are highlighted with a solid black circle. Full amino acid sequences of R2R3-MYBs were aligned using Muscle software, and phylogenetic tree was constructed with MEGA software (version 6.0) using maximum likelihood method. Numbers near branches indicate bootstrap values that were calculated from 1000 replicate analyses. The scale bar represents 0.2 substitutions per site. The accession numbers are as follows: *Arabidopsis thaliana* AtMYB4 (AT4G38620), AtMYB6 (AT4G09460), AtPAP1 (AT1G56650), AtMYB123 (AT5G35550), AtMYB32 (AT4G34990); *Vitis vinifera* VvMYBA1 (AB097923), VvMYBPA2 (EU919682), VvMYBPA1 (AM259485), VvMYBC2-L1 (JX050227); *Prunus persica* PpMYB10.2 (EU155160), PpMYBPA1 (CV047374), Peace (AB897865), PpMYB9 (KT159232), PpMYB17 (KT159233), PpMYB18 (KT159234), PpMYB19 (KT159235), PpMYB20 (KT159236), PpMYB10.4 (KF999985), PpMYB10.1 (XM_007216468); *Malus domestica* MdMYB10 (DQ267897), MdMYB16 (HM122617); *Fragaria ananassa* FaMYB9 (JQ989281), FaMYB11 (JQ989282), FaMYB1 (AF401220); *Oryza sativa* OsMYB3 (D88619); *Diospyros kaki* DkMYB4 (AB503701); *Trifolium arvense* TaMYB14 (JN049641); *Petunia x hybrid* PhAN2 (ABO21074); *Citrus sinensis* CsRuby (NM_001288889); *Lotus japonicus* LjTT2a (AB300033); *Antirrhinum majus* AmMYB308 (P81393); *Populus tremula x Populus tremuloides* PtMYB182 (KP723392); *Rosa hybrida* RhMYB10(EU155164); *Fragaria vesca* FvMYB90 like(XM_004302074); *Pyrus x bretschneideri* PbMYB208 like (XM_009339740).

Phylogenetic analysis showed that all the R2R3-MYBs except *PpMYB9* were clustered with known function MYBs of flavonoid biosynthesis (**Figure 2**). For example, *PpMYB10.2* was closely related to anthocyanin MYB activators such as *MdMYB10* and *PpMYB10.1*, while *PpMYBPA1* and *Peace* were similar to PA-related MYBs, *VvMYBPA1* and *VvMYBAP2*, respectively. *PpMYB17-20* was closely related to flavonoid MYB repressors such as *FaMYB1* and *VvMYBC2-L1*. In contrast, *PpMYB9* along with other unknown function MYBs formed a new group, which was phylogenetically related to anthocyanin MYB activators. Moreover, we further checked the *MYB9* gene in sequenced genomes of 24 plant species, and found that it is present in only seven species, including soybean, *Lotus japonicus, Medicago truncatula*, apple, *Eucalyptus grandis*, cotton, and papaya (**Table 1**).

**Table 1:** Copy numbers of *MYB9* gene in the sequenced genomes of eudicots.

Eudicots			Species	*MYB9*
Rosids	Caryophyllales	Amaranthaceae	Beet	0
	Vitales	Vitaceae	Grapevine	0
	Eudicots I	Salicaceae	Poplar	0
		Euphorbiaceae	Cassava	0
			Ricinus	0
		Fabaceae	Soybean	2
			*L. japonicus*	1
			*M. truncatula*	1
		Rosaceae	Peach	1
			Apple	1
			Strawberry	0
		Cucurbitaceae	Muskmelon	0
			Watermelon	0
	Eudicots II	Myrtaceae	*E. grandis*	1
		Rutaceae	Orange	0
		Malvaceae	Cocoa	0
			Cotton	1
		Caricaceae	Papaya	1
		Brassicaceae	*T. parvula*	0
			*B. rapa*	0
			*C. rubella*	0
			*A. lyrata*	0
			*A. thaliana*	0
Asterids		Solanaceae	Potato	0
			Tomato	0

**FIGURE 3:**
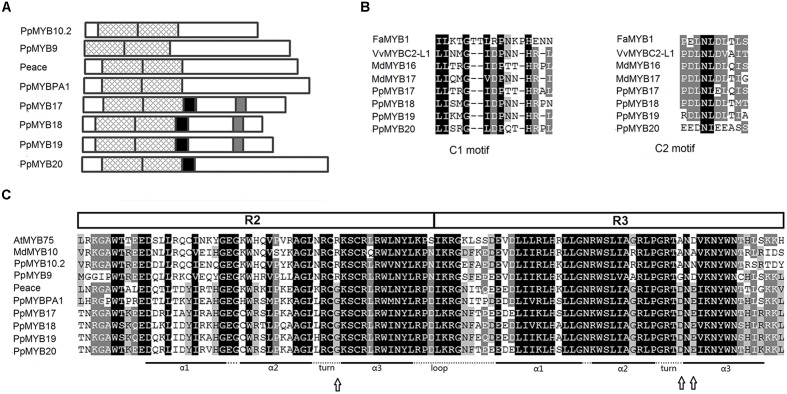
**Diagram and alignment of eight R2R3-MYB transcription factors (TFs). (A)**, structural diagram of the eight R2R3-MYBs highly expressed in peach flower. Cross lines indicate R2 and R3 SANT domains. The black and grey boxes stand for C1 and C2 motif, respectively. **(B,C)**, amino acid sequence alignment of R2R3 domain and C1/C2 motif, respectively, of the MYBs in peach and other dicots. Arrows highlight key amino acid residues that mediate promoter targets specificity for the anthocyanin and proanthocyanin (PA) pathway (Heppel et al., 2013).

Sequence alignment revealed that all the eight MYBs contained conserved R2 (53 amino acids) and R3 (52 amino acid) domains (**Figure 3A**). However, only four repressor-like MYBs, PpMYB17 through PpMYB20, contained C1 and/or C2 motifs (**Figures 3A,B**). A residue at position 39 in the R2 domain and four residues at positions 90–93 in the R3 domain are known to be key amino acid elements that control the specificity of MYB regulators for either the anthocyanin or PA pathway (Heppel et al., 2013). Both PpMYBPA1 and Peace shared the same residue Gly at position 39 in the R2 domain and Asp-Asn-Glu-Ile/Val at positions 90–93 in the R3 domain (**Figure 3C**), which suggests that they are probably involved in PA pathway. Similarly, PpMYB10.2 and PpMYB9 contained the same residue Arg at position 39 in the R2 domain, but the residus at positions 90–93 of PpMYB9 (Gly-Asn-Asp-Val) were different from that of their counterpart in PpMYB10.2 (Ala-Asn-Asn-Val), as well as other anthocyanin MYB activators such as MdMYB10 and AtMYB75. In addition, the residue at position 99 was also different between PpMYB9 (Cys) and other anthocyanin MYB activators (Thr or Ser). These results suggest that the recognition site of *PpMYB9* in the promoter of anthocyanin structural genes may differ from that of other anthocyanin MYB activators, leading to functional divergence between *PpMYB9* and *PpMYB10.2*.

### Functional Analysis of the Eight R2R3-MYBs Using Dual Luciferase Assay

**FIGURE 4:**
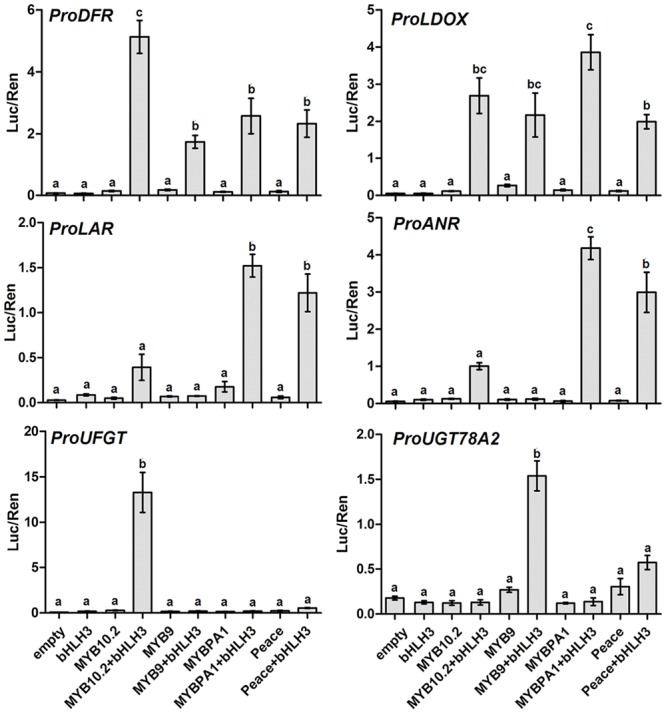
**Estimation of effect of different R2R3-MYBs with PpbHLH3 partner on activation of the promoter of anthocyanin or PA pathway genes using transient dual luciferase assay.** Promoter activity was shown as the ratio of firefly Luciferase (Luc) activity to Renilla luciferase (Ren) activity. Different lowercase letters indicate the significant differences among treatments by Tukey’s test at *P* < 0.01. Error bars show SE of four biological replicates.

To testify whether the eight R2R3-MYBs had direct activation on transcription of structural genes, the promoter regions of six flavonoid pathway genes in peach, *DFR, LDOX, LAR1, ANR, UFGT*, and *UGT78A2*, were isolated and inserted into dual luciferase assay system (Hellens et al., 2005). Four *MYB* genes (*PpMYB10.2, PpMYB9, PpMYBPA1*, and *Peace*) with the *PpbHLH3* partner were all able to activate the promoters of early flavonoid pathway genes, with LUC/REN ratio ranging from 1.7 to 5.1 for the *DFR* promoter, and from 1.9 to 3.8 for the *LDOX* promoter (**Figure 4**). This suggests that these four *MYB* genes are probably involved in the regulation of anthocyanin or proanthocyanin biosynthesis in peach.

Our previous study shows that cyanidin 3-glucoside is the main component of anthocyanins in peach flower, and two *UFGT78* genes, *PpUGT78B* (also commonly called *PpUFGT* in previous studies) and *PpUGT78A2*, are involved in the glycosylation of cyanidin (Cheng et al., 2014). Here, dual luciferase assay further revealed that only *PpMYB10.2* co-infiltrated with *PpbHLH3* was able to activate the *PpUFGT* promoter, with a LUC/REN ratio of 13.3. However, either *PpMYB10.2* or *PpMYBPA1* co-infiltrated with *PpbHLH3* showed a weak activation on the *PpUFGTA2* promoter. The *PpMYB9* gene with the *PpbHLH3* partner showed the strongest activation on the *PpUFGTA2* promoter, with a LUC/REN ratio of 1.5. In addition, *Peace* with the *PpbHLH3* partner showed a twofold decrease in the *PpUFGTA2* promoter activation when compared with *PpMYB9*, but its activity was approximately four or fivefold higher than that of *PpMYBPA1*.

In peach, two structural genes, *PpLAR1* and *PpANR*, were found to be related to proanthocyanidin biosynthesis (Ravaglia et al., 2013). Here, our results indicated that both *PpMYBPA1* and *Peace* with the *PpbHLH3* partner displayed a strong activation on the *PpLAR1* promoter, with the LUC/REN ratios of 1.5 and 1.2, respectively. Similarly, both *PpMYBPA1* and *Peace* with the *PpbHLH3* partner were also able to effectively activate the *PpANR* promoter, with the LUC/REN ratios of 4.2 and 3.0, respectively. *PpMYB10.2* co-infiltrated with *PpbHLH3* showed a slight activation on the promoters of *PpLAR1* and *PpANR*, with the LUC/REN ratios of 0.4 and 1.0, respectively. However, no activation was observed for the infiltration of *PpMYB9* alone or with the *PpbHLH3* partner.

**FIGURE 5:**
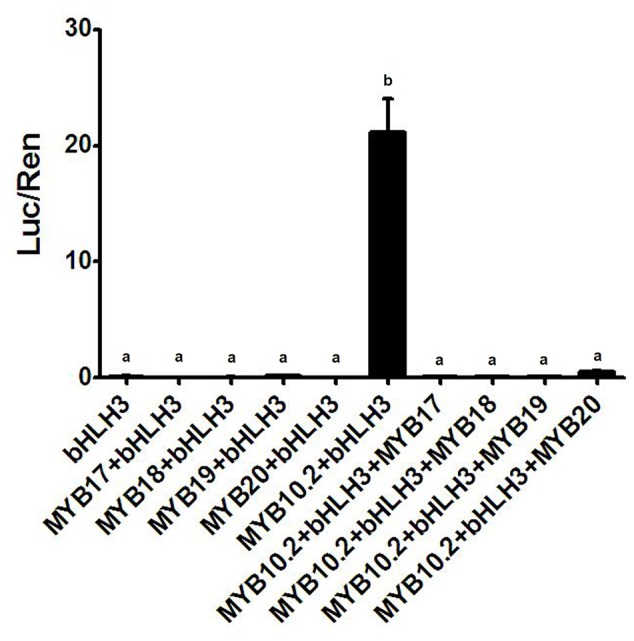
**Validation of activation effect of four R2R3-MYBs on the *PpDFR* promoter using dual luciferase assay.** Different lowercase letters indicate the significant differences among treatments by Tukey’s test at *P* < 0.001. Results represent the means of four biological replicates.

In addition, *PpDFR* promoter was selected to validate the function of the four flavonoid MYB repressors, *PpMYB17-20*. Co-infiltration of *PpMYB10.2* and *PpbHLH3* showed a strong activation on the *DFR* promoter (**Figure 5**). However, this activation was completely counteracted by addition of any of the four flavonoid MYB repressors.

In general, the above results suggest that of the eight R2R3-MYBs expressed in peach flower, *PpMYB10.2* and *PpMYB9* are anthocyanin-related MYB activators, while *PpMYBPA1* and *Peace* belong to proanthocyanin-related MYB regulators. However, the other four, *PpMYB17-20*, are flavonoid-related MYB repressors.

### Transient Color Assays of the Peach *MYB9* Activity

**FIGURE 6:**
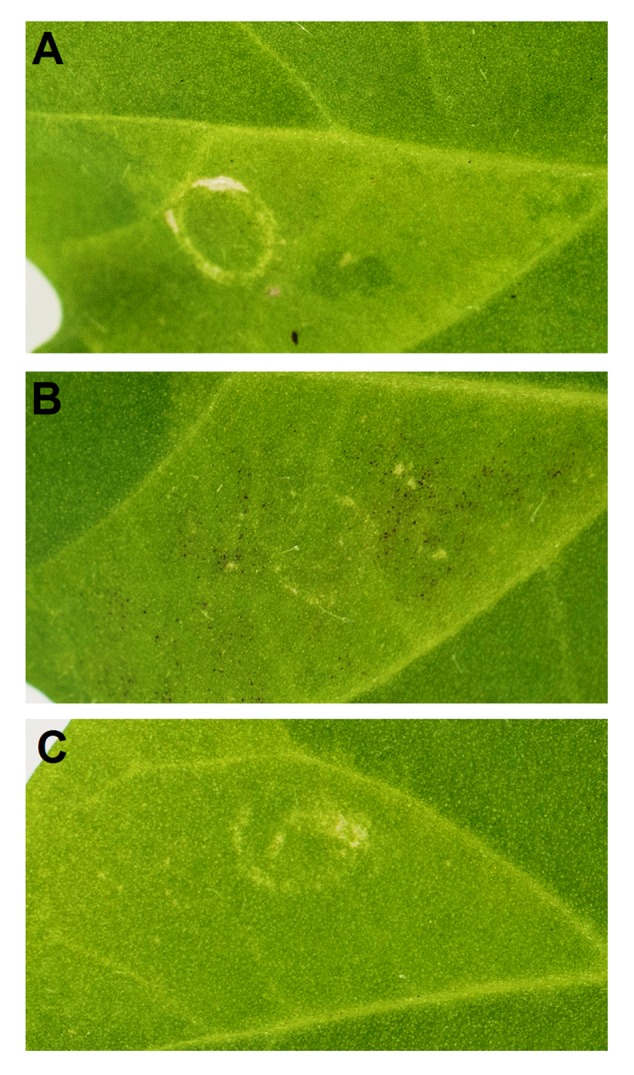
**Transient color assay of the *PpMYB9* activity in *N. tabacum* leaf. (A–C)** indicate infiltration with *PpMYB9*+*PpbHLH3, PpMYB9*+*PpbHLH3*+*proPpUGT78A2*::*PpUGT78A2*, and *proPpUGT78A2*::*PpUGT78A2*, respectively. Photos were taken 2 weeks after infiltration.

Since *PpMYB9* represents a new MYB group as mentioned above, its function was further validated by transient color assay in *N. tabacum* leaf via *Agrobacterium* infiltration. Initially, *PpMYB9* and *PpbHLH3* were infiltrated into the abaxial side of tobacco leaf. However, no red pigmentation was observed in 2 weeks (**Figure 6A**). Considering the fact that *PpMYB9* specifically activated the promoter of *PpUGT78A2* instead of the common *PpUFGT*, a construct proPpUGT78A2::PpUGT78A2 was co-infiltrated with *PpMYB9* and *PpbHLH3*. Red pigmentation was detected around the infiltration site in 2 weeks (**Figure 6B**). However, no pigmentation was observed for the infiltration of proPpUGT78A2::PpUGT78A2 alone (**Figure 6C**). These results indicated that heterologous expression of *PpMYB9* and *PpbHLH3* was able to induce anthocyanin accumulation via activating transcription of *PpUGT78A2*.

### Expression Profiles of the Eight R2R3-MYBs in Peach Flower

**FIGURE 7:**
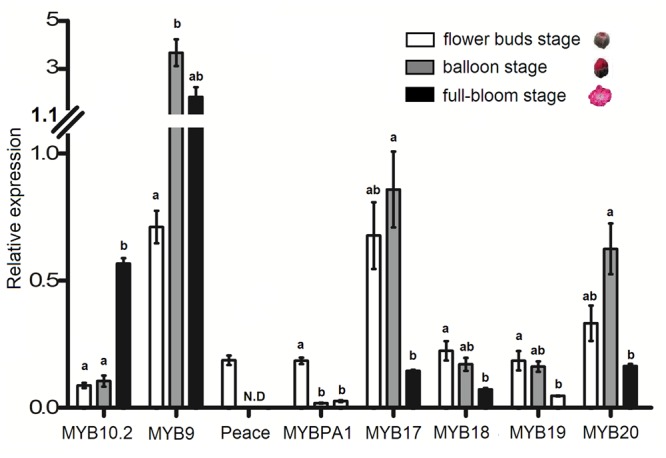
**Expression profiles of the eight R2R3-MYBs in flowers of cv. Mantianhong at different developmental stages.** Different lowercase letters indicate the significant differences among treatments by Tukey’s test at *P* < 0.01. Error bars show SE of the means of three biological replicates.

An ornamental peach variety ‘Mantianhong’ was selected to investigate the expression profile of the eight R2R3-MYBs in flower. Of the four MYB activators, *PpMYB9* showed the highest level of expression throughout the developmental stages analyzed (**Figure 7**). The transcript level of *PpMYB10.2* increased significantly during the process of flower development, with a high peak at full-bloom stage. Both *PpMYBPA1* and *Peace* were expressed at bud stage, whereas, with almost undetectable transcript level at either balloon or full-bloom stages. Of the four MYB repressors, *PpMYB17* and *PpMYB20* were highly expressed at bud and bloom stages, but decreased significantly at full-bloom stage. The transcript level of *PpMYB18* and *PpMYB19* was relatively low and showed a significantly decreasing pattern during the process of flower development.

## Discussion

### Anthocyanin Accumulation in Peach Flower Is Regulated by a Set of R2R3-MYB Genes

In plants, anthocyanin biosynthesis is regulated by the MYB-bHLH-WD40 “MBW” complex. Of these three regulators, R2R3-MYBs play a crucial role in determining anthocyanin accumulation (Ramsay and Glover, 2005). It has been documented that multiple R2R3-MYB TFs are involved in the regulation of floral pigmentation in ornamental plants. For example, four R2R3-MYB TFs, *AN2, AN4, PHZ*, and *DPL*, control anthocyanin accumulation in different parts of petunia flower (Quattrocchio et al., 1998, 1999; Albert et al., 2011). Three R2R3-MYB TFs, *Rosea1, Rosea2*, and *Venosa*, are involved in the regulation of flower pigmentation in the genus *Antirrhinum* (Schwinn et al., 2006). Three R2R3-MYB TFs are responsible for distinct patterns of floral pigmentation in Orchidaceae (Hsu et al., 2015).

In this study, four R2R3-MYB activators, *PpMYB9, PpMYB10.2, PpMYBPA1*, and *Peace*, were found in peach flower transcriptome. Functional analysis demonstrated that *PpMYB10.2* and *PpMYB9* are related to anthocyanin biosynthesis, whilst *PpMYBPA1* and *Peace* are mainly involved in regulation of PA biosynthesis. Both *PpMYB10.2* and *PpMYB9* are expressed in flower, with a high-level expression in late development stage. This suggests that *PpMYB9* and *PpMYB10.2* are crucial for floral coloration in peach. The high-levels of expression of these two *MYB* genes during might play an important role in maintenance of the flower coloration during late stages of flower development. Unlike the anthocyanin-related MYB activators, *PpMYBPA1* and *Peace* show extremely low or undetectable levels of expression in flower at balloon and full-bloom stages, suggesting that they may play little role in peach floral coloration at the later developmental stages. A more recent study shows that *Peace* has high-level expression in flower bud of cv. Genpei and its expression change causes variegated pattern of floral coloration (Uematsu et al., 2014). Interestingly, *Peace* was shown to activate anthocyanin biosynthesis. However, our study indicates that *Peace* is phylogenetically related to *PpMYBPA1*, and both of them have a strong activation on the promoters of PA biosynthetic genes, *PpLAR* and *PpANR.* Overexpression of either *LAR* or *ANR* can result in loss of anthocyanin in flower (Han et al., 2012; Liao et al., 2015). Thus, the regulatory role of *Peace* in peach flower coloration needs further investigation.

It is worth noting that *Peace* belongs to PA regulator group, and its transient expression was shown to induce pigmentation in white flower of ‘Genpei’ (Uematsu et al., 2014). However, our study demonstrates that the *Peace* gene can efficiently activate PA-specific pathway genes such as *LAR* and *ANR*, whereas, with low activation of anthocyanin-specific pathway genes such as *PpUGT78A2*. This indicates that the *Peace* gene is unlikely to play an important role in anthocyanin pigmentation. More studies are still needed to address the mechanism by which the *Peace* gene regulates anthocyanin pigmentation in peach flower.

Besides anthocyanin-related MYB activators, four flavonoid-related MYB repressors, *PpMYB17* through *PpMYB20*, are also found in peach flower transcriptome. All these MYB repressors have a strong repressive effect on flavonoid pathway genes such as *DFR*, and are expressed in flower during the whole process of development. These results indicate that *PpMYB17-20* may play a negative role in the regulation of anthocyanin accumulation in peach flower.

Taken together, our study suggests that anthocyanin accumulation in peach flower is regulated by a set of R2R3-MYB genes, including four MYB activators and four MYB repressors. Coordinate regulation of multiple R2R3-MYB genes is likely responsible for flower color intensity variation in peach, ranging from white or pale to red, pink, and purple.

### *PpMYB9* Represents a New Group of R2R3-MYB Genes Regulating Anthocyanin Biosynthesis in Plants

In this study, phylogenetic analysis reveals that anthocyanin-related MYB activators are divided into two groups, MYB10 and MYB9 (**Figure 2** and Supplementary Figure S1). The MYB10 group consists of the peach *PpMYB10.2* and most previously reported R2R3-MYB activators controlling anthocyanin pigmentation in dicots, such as *Arabidopsis AtPAP1* and *AtPAP2* (Borevitz et al., 2000), apple *MdMYB10* (Espley et al., 2007), citrus *CsRuby* (Butelli et al., 2012), *Petunia × hybrid PhAN2* (Quattrocchio et al., 1998), *AmVENOSA, Antirrhinum majus AmROSEA1* and *AmROSEA2* (Schwinn et al., 2006), and grapevine *VvMYBA1* and *VvMYBA2* (Walker et al., 2007).

The MYB9 group that includes the peach *PpMYB9* is different from the MYB10 group. Firstly, the *MYB9* gene is lost in most dicot species (**Table 1**), whereas, the *MYB10* gene is present in all eudicot species. This suggests that the *MYB10* group represents a common anthocyanin-related MYB activator in plants, while the MYB9 group is a rare type of anthocyanin-related MYB activator and only present in a limited number of plant species. Secondly, all previously reported *MYB10* genes, such as *AtPAP1*/*AtMYB75* (Borevitz et al., 2000), *MdMYB10* (Espley et al., 2007), *PpMYB10.1* (Zhou et al., 2015b), and *VvMYBA1* (Walker et al., 2007), show a strong activation effect on the *UFGT* promoter. In contrast, the peach *PpMYB9* is unable to activate the *PpUFGT* promoter, but it shows a strong activation on the *PpUGT78A2* promoter. Moreover, the *PpMYB10.2* gene, a member of the MYB10 group, displays no activation effect on the *PpUGT78A2* promoter. These results indicate that divergence in target genes has occurred between the MYB9 and MYB10 activators. Thirdly, two previously reported *MYB10* genes, *PpMYB10.1* (Rahim et al., 2014; Zhou et al., 2015b) and *PpMYB10.4* (Zhou et al., 2014) are related to anthocyanin pigmentation in fruit and leaf of peach, respecitvely. Here, our study reveals that an additional *MYB10* gene *PpMYB10.2* is involved in peach flower coloration. This suggests that the *MYB10* gene has undergone duplication and subsequent divergence in spatial expression patterns. However, *PpMYB9* is exclusively expressed in flower of peach (**Figure 1**), and its expression profile is different from those of the *MYB10* genes in peach.

Taken together, the above results suggest that the *PpMYB9* gene represents a novel group of anthocyanin-related MYBs, which may have diverged in function from the *MYB10* genes in plants. In addition, it is worth noting that a R2R3-MYB gene in rose, designated *RhMYB10*, was reported in a previous study (Lin-Wang et al., 2010). *RhMYB10* is able to activate transcription of *Arabidopsis DFR* gene when it was co-transformed with bHLHs. *RhMYB10* was classified as a *MYB10* gene although it represents an outgroup of the *MYB10* genes in the phylogenetic tree (Lin-Wang et al., 2010). Here, our study indicates that *RhMYB10* is closely related to *PpMYB9*, and should be grouped into the MYB9 group. This finding will be useful for both identification of the *MYB9* gene in other species and pursing a comprehensive understanding of the evolutionary process of R2R3-MYB genes in plants.

## Author Contributions

YH and HZ conceived and designed the experiments. HZ, QP, LL, LW, and XD performed the experiments. YH and HZ wrote the paper. JZ, AO, FR, and QJ revised the manuscript.

## Conflict of Interest Statement

The authors declare that the research was conducted in the absence of any commercial or financial relationships that could be construed as a potential conflict of interest.
