# Supplementary material for: Multiple R2R3-MYB Transcription Factors Involved in the Regulation of Anthocyanin Accumulation in Peach Flower
Source: Front Plant Sci. 2016 Oct 21;7:1557. doi: 10.3389/fpls.2016.01557 (PMC5073212; doi:10.3389/fpls.2016.01557)
Supplement: Supplementary file 1 [file Table_1.DOC]

Table S1. Sequences of primers used for gene cloning and vectors construction

| Primer name | Sequence | Description |
| --- | --- | --- |
| PpMYB9OEF | CCGGAATTCATGGGTGGCATTCCATGG | Primers used for cloning and insertion of PpMYB9 into vector pSAK277 |
| PpMYB9OER | CCCAAGCTTTTAACAATGAGTCCAGAGGTCCAC |
| PpMYBPA1OEF | CCCAAGCTTATGGGAAGGGCTCCTTGTTG | Primers used for cloning and insertion of PpMYBPA1 into vector pSAK277 |
| PpMYBPA1OER | GCTCTAGATTATATCAGCAGTGACTCAGCAAATG |
| PeaceOEF | CCCAAGCTTATGGGAAGAACTCCTTGTTGCT | Primers used for cloning and insertion of PpPeace into vector pSAK277 |
| PeaceOER | GCTCTAGATTAATGGTGGAAACAGTCATCATCT |
| PpMYB10.2OEF | CCGGAATTCATGGAGGGTTATGACTTGAGTG | Primers used for cloning and insertion of PpMYB10.2 into vector pSAK277 |
| PpMYB10.2OER | CCGCTCGAGTTACTTTCTATATTCTTCATTTGAATGA |
| PpMYB17OEF | CCGCTCGAGATGGGAAGATCTCCTTGCTGTG | Primers used for cloning and insertion of PpMYB17 into vector pSAK277 |
| PpMYB17OER | GCTCTAGATCATTTCATCTCCAAGCTTCTGTAA |
| PpMYB18OEF | CCGCTCGAGATGAGGAAACCCTGCTGCGA | Primers used for cloning and insertion of PpMYB18 into vector pSAK277 |
| PpMYB18OER | GCTCTAGATCAAGAGAGGAAAGATATTGGTGGA |
| PpMYB19OEF | CCGCTCGAGATGAGAAAACCTTGCTGTGAAAAAG | Primers used for cloning and insertion of PpMYB19 into vector pSAK277 |
| PpMYB19OER | GCTCTAGATTATCTAAAAAGAGCAAGAGTGGGTGA |
| PpMYB20OEF | CCGCTCGAGATGGGGAGGTCACCTTGCTG | Primers used for cloning and insertion of PpMYB20 into vector pSAK277 |
| PpMYB20OER | GCTCTAGACTATGAATTCAAAGGTCTGTGAAATCT |
| PpbHLH3topoF | CACCATGGCTGCACCGCCAAGT | Primers used for cloning of PpbHLH3 |
| PpbHLH3topoR | CTAGGAATCAGATTGGGGAATTATT |  |
| proLDOX-LUCF | CGCGGATCCAATGGAGTTACCCACAGATTTCG | Primers used for cloning and insertion of pro*LDOX* into vector pGreenII LUC+ |
| proLDOX-LUCR | TCACCATGGTGGCAGCCGGCTCTTC |
| proLAR1-LUCF | CGCGGATCCGGTGCTGATGATCAATGACTGC | Primers used for cloning and insertion of pro*PpLAR1* into vector pGreenII LUC+ |
| proLAR1-LUCR | CATGCCATGGCTGGCTGCTGGCT |
| proANR-LUCF | CCCCCCGGGCGCTGTTATGGAAAGGGTCACT | Primers used for cloning and insertion of pro*PpANR* into vector pGreenII LUC+ |
| proANR-LUCR | GCTGTCTTCTTTGAGATGGGTTG |
| proDFR-LUCF | CGCGGATCCGAATGCACTACTGGAACCGACTG | Primers used for cloning and insertion of pro*PpDFR* into vector pGreenII LUC+ |
| proDFR-LUCR | CATGCCATGGTTGAATCAAATCAAGTATGTAC |
| proUFGTF | CTGTGCCGCAATATCTGACATC | Primers used for cloning of pro*PpUFGT* |
| proUFGTR | TATGAGCTAATAAGACTAATTGGAGTGG |  |
| proUGT78A2F | CCCCCCGGGACGAGGATGTCGAGCACATAAG | Primers used for cloning and insertion of pro*PpUGT78A2* into vector pGreenII LUC+ |
| proUGT78A2R | TCCTGCCATGGTTGTTAGATGG |
