# Supplementary material for: Multiple R2R3-MYB Transcription Factors Involved in the Regulation of Anthocyanin Accumulation in Peach Flower
Source: Front Plant Sci. 2016 Oct 21;7:1557. doi: 10.3389/fpls.2016.01557 (PMC5073212; doi:10.3389/fpls.2016.01557)
Supplement: Supplementary file 2 [file Table_2.DOC]

Table S2. Sequences of primers used for qRT-PCR analysis in peach

| Gene | Gene number | Forward (5’-3’) | Reverse (5’-3’) |
| --- | --- | --- | --- |
| PpMYB10.2 | ppa016711m | AAGGCCACAACCAAGAAGA | AACCCAAGACCAGAACCTGT |
| PpMYB9 | ppa010069m | GCTCCTAGGGAATAGGTGGTCA | TGCACAATGCTTGTTCTTGTCTT |
| Peace | ppa023768m | GCTATTAGGCAACAGATGGTCAC | GAGGATTGCTTTGTTTGTTGATC |
| PpMYBPA1 | ppa009439m | GGGAAGGCCATTGGAGATC | AACGGTTGCCCAGAAGTGA |
| PpMYB17 | ppa010277m | GTTGCAGGCTGCGTTGGA | CGAGGAGGCTATGGAGTTTGAT |
| PpMYB18 | ppa010846m | GATAAACTATCTACGGCCAGACCT | GGCAATCTCCCAGCAATCA |
| PpMYB19 | ppa010716m | GAGGACGGACAACGAGGTGA | TTGAGGATTCGGACGAGGAA |
| PpMYB20 | ppa008906m | ACAACAGCAACAGCAGAAATTG | GAGTTGGCTCGGACTGGAAG |
| PpTEF2 | ppa001368m | GGTGTGACGATGAAGAGTGATG | TGAAGGAGAGGGAAGGTGAAAG |
