# Supplementary material for: Multiple R2R3-MYB Transcription Factors Involved in the Regulation of Anthocyanin Accumulation in Peach Flower
Source: Front Plant Sci. 2016 Oct 21;7:1557. doi: 10.3389/fpls.2016.01557 (PMC5073212; doi:10.3389/fpls.2016.01557)
Supplement: Supplementary file 3 [file Image_1.PDF]

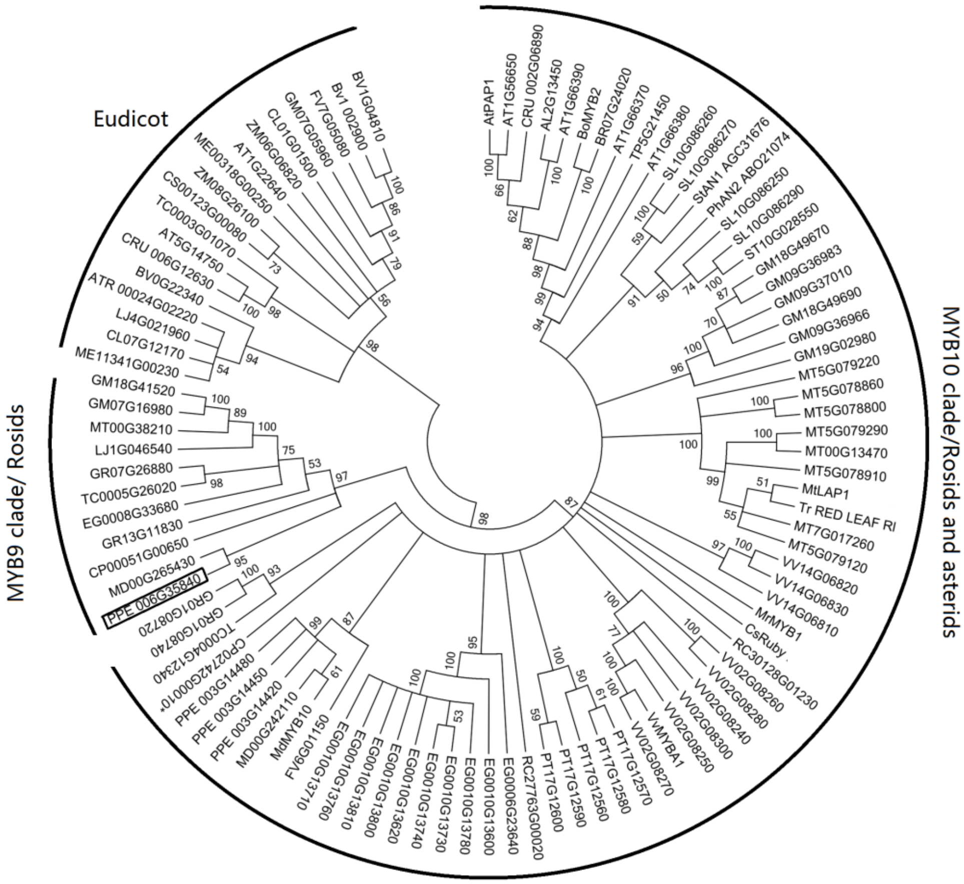

Figure S1 Phylogenetic analysis of R2R3-MYB genes from different plant species.
